# Test-Time Augmentations and Quality Controls for Improving Regional Seismic Phase Picking

**DOI:** 10.3390/s25237238

**Published:** 2025-11-27

**Authors:** Bingyao Han, Lin Tang, Li Ma, Hua Kong, Zhuowei Xiao

**Affiliations:** 1 Key Laboratory of Deep Petroleum Intelligent Exploration and Development, Institute of Geology and Geophysics, Chinese Academy of Sciences, Beijing 100029, China; hanby@mail.iggcas.ac.cn; 2 Sichuan Earthquake Administration, Chengdu 610040, China; 3 Liaoning Earthquake Administration, Shenyang 110034, China; rongcuoxue@163.com; 4 Key Laboratory of Planetary Science and Frontier Technology, Institute of Geology and Geophysics, Chinese Academy of Sciences, Beijing 100029, China; konghua21@mails.ucas.ac.cn; 5 University of Chinese Academy of Sciences, Beijing 101408, China

**Keywords:** seismic phase picking, deep learning, test-time augmentation

## Abstract

**Highlights:**

**What are the main findings?**

- Test-time augmentations could enhance the reliability of seismic first-arrival picking.
- Results obtained under different augmentations can serve as an effective quality control indicator.

**What is the implication of the main finding?**

- The combination of test-time augmentation and quality control improves the reliability of regional seismic phase picking
- Our findings offer practical guidance for deploying deep-learning-based pickers for identifying regional seismic phases.

**Abstract:**

Regional seismic phases are essential for imaging Earth’s internal structure. Although extensive regional seismic networks are publicly available worldwide, only a small fraction of recorded phase arrivals are picked for constraining earthquake source parameters, leaving most data untapped. Recent deep-learning methods offer powerful tools for automatic phase picking, yet their performance often lags behind that of human experts, particularly at relatively large epicentral distances such as the case of the Pn phase (~200–2000 km). Here, we systematically assess the effect of different test-time augmentation strategies on the Pn phase picking performance using PickNet and PhaseNet, along with the Seis-PnSn dataset containing data worldwide to simulate the out-of-distribution situation. We also propose quality control measures to obtain reliable results when ground truths are unknown. Our experiments show that filter-bank augmentation is more effective than the shift augmentation and the rotation augmentation, improving the proportion of picks within ±0.5/1.0 s errors to 53.87%/70.82% compared with the baseline of 48.98%/66.94% for PickNet and ±0.5/1.0 s errors to 48.45%/67.06% compared with the baseline of 46.32%/64.28% for PhaseNet. After the quality control using the standard deviation of different augmentation results, the proportion is further boosted to 67.39%/78.53% for PickNet and 57.99%/74.72% for PhaseNet. Additionally, we provide the workflow in our study as scripts for real-world data processing. Our work enhances both the accuracy and accessibility of regional seismic phase picking, thereby contributing to the studies of Earth’s internal structure and earthquake source characterization.

## 1. Introduction

Regional seismic phases recorded by global seismic networks are indispensable for rapid earthquake response, source characterization, and imaging of Earth’s interiors [1]. Seismic phases also contribute to the understanding of earthquake nucleation and rupture processes [2], the revealing of previously unmapped faults and detailed fault geometry [3], the evaluation of induced seismicity [4], the continental collision [5], etc. Yet only a small fraction of available seismic arrivals are routinely picked, leaving most continuous waveform data underused. Recent growth in open benchmarks [6] and datasets [7,8,9,10], together with advances in deep-learning detectors and pickers [11,12,13,14,15,16], has allowed automated, petabyte-scale seismicity analysis that yields billions of high-quality phase picks [17], which is promising for advancing seismology. To provide a numerical example of how much information on continuous waveforms remains underutilized, in [13], for the same 300 events, the PickNet model obtained 97,998 P-wave and 92,229 S-wave arrival times recorded at 782 Hi-net stations. In contrast, the JMA catalog provided only 13,765 P-wave and 8643 S-wave arrival times for the same earthquakes, recorded at 1246 seismic stations (including 782 Hi-net stations and 464 stations from other local networks).

A vibrant ‘ecosystem’ of artificial intelligence for earthquake seismology has emerged spanning million-scale seismic datasets (e.g., STEAD [7], INSTANCE [8], MLAAPDE [10], DiTing [9], CREW [18], Seis-PnSn [19]), learning architectures (e.g., PhaseNet [11], EqTransformer [12], PickNet [13]), and end-to-end workflows (e.g., LocFlow [20], QuakeFlow [21]). Although augmentations are well studied for training the deep-learning pickers [22], little attention has been paid to inference-time strategy, which focuses on how to deploy trained models so that predictions are both accurate and reliable at different scales [12,23,24]. This omission matters because modern models can produce enormous numbers of candidate picks. Also, effective quality control is crucial for downstream scientific tasks. In addition, most of the current deep-learning pickers are designed for local networks and distances, while robust, user-friendly solutions that perform reliably at regional scales (epicentral distances up to ~18°) are relatively few.

Test-time augmentation (TTA) is a strategy in which, during inference, multiple augmented versions of the input are generated, and their predictions are averaged to improve model performance [25]. Earlier studies have implemented TTA by keeping dropout active at inference time to obtain Monte Carlo estimates [12,24] or by rescaling input data to accommodate earthquakes at quite different scales [23]. Together, these works underscore the importance of TTA. In this paper, we focus on test-time improvement of deep-learning pickers by systematically evaluating TTA within an open-source implementation of the PickNet model [13] as well as the PhaseNet model [11]. We selected these two models because they directly support studies on seismic tomography and seismicity [26,27,28,29]. One limitation of the PickNet model is that PickNet requires theoretical travel times to define the input time windows, so it is most suitable for regional phase picking when an earthquake catalog is available. Please note that the advantage of deep-learning methods over previously known methods, such as STA/LTA, has been demonstrated in many earlier studies [12]. In this work, we focus on evaluating the effectiveness of TTA and therefore do not compare against non-deep-learning methods. Experiments are conducted on the newly compiled Seis-PnSn dataset [19] comprising ~1.3 million three-component seismograms recorded from 2000 to 2019 at epicentral distances of 1.8–18°. We investigate three complementary TTA modules: (i) random temporal shifts, (ii) multi-band frequency filtering, and (iii) rotation augmentation. We further introduce a quality control (QC) method based on the consistency of the picks from different augmentations.

Relative to a no-augmentation baseline, in which 48.98%/66.94% of picks fall within ±0.5 s/±1.0 s of analyst manual picks for PickNet and 46.32%/64.28% for PhaseNet, TTA yields incremental gains. Filter-bank augmentation increases these proportions to 52.64%/77.61% for PickNet and 48.45%/67.06% for PhaseNet, while random-shift augmentation achieves 54.99%/70.08% for PickNet and 46.50%/53.42% for PhaseNet. After applying our QC workflow, performance further improves to 63.92%/86.40% (filter-bank plus QC) and 70.07%/88.46% (random-shift plus QC) for PickNet and 54.34%/73.08% (filter-bank plus QC) and 53.42%/71.89% (random-shift plus QC) for PhaseNet. These results demonstrate that simple, principled test-time strategies, coupled with lightweight QC, can enhance regional phase picking results. We release easy-to-use Python scripts (10.5281/zenodo.17303467) with demonstrative examples for processing real-world data. By combining test-time augmentation with practical QC and packaging, our work provides a beneficial reference for automated regional Pn phase picking.

## 2. Materials and Methods

### 2.1. Deep Learning Models

The PickNet model [13] is a fully convolutional network built on a modified VGG-16 backbone augmented with Rich Side-output Residual Networks [30,31], where side-output layers after each convolutional block extract multi-scale features that are fused via residual units and supervised at every level to learn the picking task hierarchically.

In this study, we use a customed open-source version of PickNet. Different from the original version that takes the vertical component as input for the P wave, this version takes a three-component waveform slice of fixed length T=3200 samples with channels {Z,N,E}. Let $X\in R^{T\times 3},X_{t,c}\;\mathrm{for}\;t\in \{1,\ldots ,T\},\;c\in \{Z,N,E\}.$ The pretrained picker is a deep network $F\left(\;\cdot \;;\theta \right)$ that outputs a per-sample pick probability trace $p\left(t\right)\in$ [0, 1] (one prediction value per sample). Without any test-time augmentation and quality control, the baseline prediction can be written as

(1) $$\begin{array}{c} p^{ori}\left(t\right)=F\left(X;\theta \right),\;\;\hat{\tau^{ori}}=\arg\underset{t\in \{1,\ldots ,T\}}{\max}p^{ori}\left(t\right). \end{array}$$

We make two main modifications to the original PickNet. First, we change the input from a single channel to three channels (Z, N, and E) to handle three-component waveform data and to support rotation-based data augmentation. Second, we increase the input window length from 1200 to 3600 samples to cover a longer time window around the seismic phase. This version is trained on data from the DiTing dataset [9] that does not overlap with the Seis-PnSn dataset, making it suitable to evaluate performance improvement under out-of-distribution cases that widely exist in practice. The PhaseNet model is implemented using the original weights provided in SeisBench [6]. For Pn phase picking, the input window and post-processing procedures are identical to those used for the PickNet model in this study.

### 2.2. Seis-PnSn Dataset

We use the test set of the Seis-PnSn dataset [19] to evaluate our method. The Seis-PnSn dataset is a global, million-scale benchmark for deep-learning-based Pn and Sn phase picking: drawing on high-quality travel-time picks from the International Seismological Centre (2000–2019) and three-component waveforms from FDSN data centers, it covers 89,297 Pn events (1,144,094 seismograms) recorded by 3662 stations, all at epicentral distances of 1.8–18°. Pn arrivals exhibit a roughly uniform distance distribution with travel times beyond 250 s. The Pn test subset used in this study comprises 180,000 waveforms. All phase picks are manually reviewed within the ISC-EHB procedure, ensuring consistently high pick quality. In addition, Seis-PnSn provides rich metadata, including detailed event and station parameters, epicentral distance, azimuth, and SNR, enabling flexible data selection and quality control. Owing to its scale, global coverage, and careful curation, Seis-PnSn can serve as a robust benchmark for developing and assessing deep-learning methods for regional Pn phase picking.

### 2.3. The Proposed Workflow

**Figure 1:**
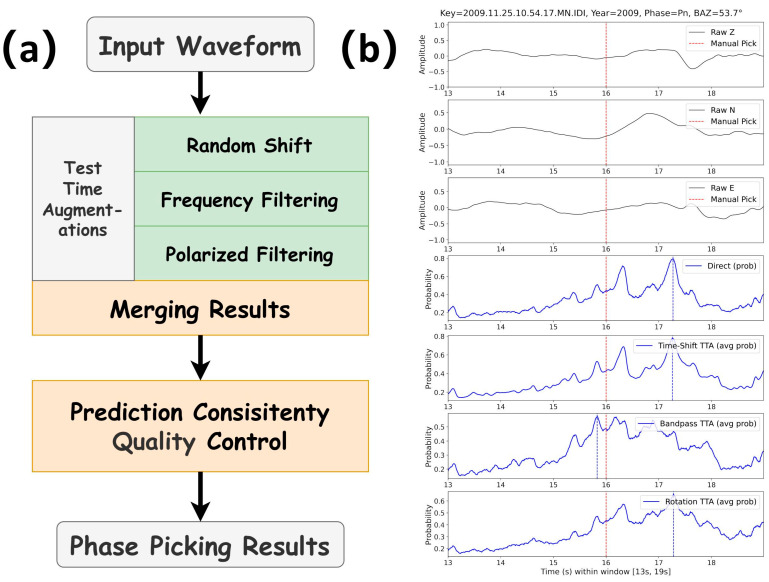
Test-time augmentation and quality control workflow with an example record. (**a**) Schematic of the inference pipeline. (**b**) Example Pn record with the analyst pick marked by the red dashed line. The lower panels show the pick-probability traces for the direct inference, time-shift TTA (averaged after realignment), band-pass/filter-bank TTA (averaged across different frequency bands), and rotation TTA (averaged across rotations). Traces are evaluated in a 13–19 s window at 100 Hz sampling.

Our workflow comprises two sequential modules, test-time augmentation (TTA) and quality control (QC) (Figure 1a). An example of output probabilities with different TTAs is shown in Figure 1b. Figure 1b illustrates these steps for a representative Pn arrival. The top three panels show the raw Z, N, and E components, with the manual pick marked by a red dashed line. The bottom four panels display the picker output probabilities for the direct prediction and for the time-shift, band-pass, and rotation TTAs as shown in Figure 1a, respectively, where the blue curves indicate the average probability over corresponding augmentations and the vertical blue line marks the automatic pick.

In the TTA module, we apply temporal jitter augmentation via predefined sample offsets; after inference, each probability trace is shifted back (inverse jitter) to the original time index, and the averages are computed on these realigned traces over the intersection of valid indices. The pre-defined shifts are Δ = {−150, −125, −100, −50, −25, −5, 0, 5, 25, 50, 100, 125, 150} samples. Let $J_{\delta }$ denote the time-shift operator by adding δ to the input sequence; the temporal jitter augmentation can be expressed as

(2) pδt=FJδX;θ, pjit¯t=1Δ∑δ∈Δpδt − δ, τjit^=arg maxt pjit¯t.

**Figure 2:**
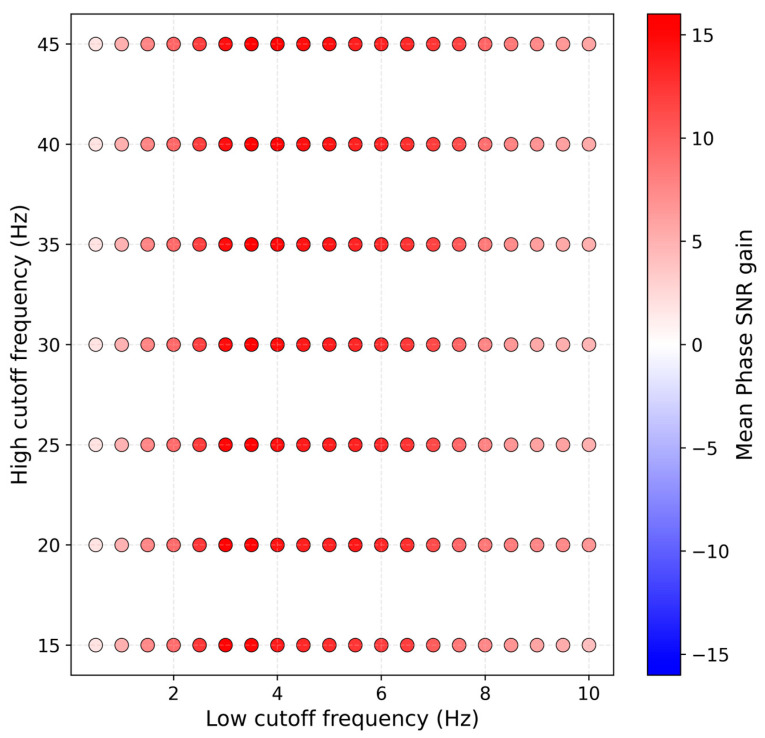
Grid search for band-pass filters based on the SNR of the Pn onset. Each dot represents one combination of low-cut and high-cut frequencies, and the color indicates the mean SNR gain (filtered minus unfiltered) averaged over 1000 sampled waveforms.

We then band-pass filter the input using a bank of frequency bands. There are two recommended aspects for choosing these pre-defined filters. The first is to design manually by specifying frequency ranges based on their experience with the target phase at given epicentral distances. The second is to randomly sample waveforms, apply a grid of candidate band-pass filters, and measure the change in signal-to-noise ratio (SNR) of the onset before and after filtering. Here, the SNR of the seismic phase onset is defined similarly to that in [32] as the ratio between the peak absolute amplitudes in the 2 s after and the 2 s before the target pick. The mean SNR gain is defined as the SNR after filtering minus the SNR before filtering. In our implementation, we randomly selected 1000 waveforms and searched over low-frequency cutoffs from 0.5 to 10 Hz with a step of 0.5 Hz, and high-frequency cutoffs from 15 to 45 Hz with a step of 5 Hz. The results of this search are shown in Figure 2. As can be seen, for low-cutoff frequencies in the range 0.5–10 Hz and high-cutoff frequencies in the range 15–45 Hz, most filter combinations increase the SNR of the Pn onset in these extracted waveforms. Based on manual expert as well as grid search results, we selected the eight filters $B=\{\left(1,40\right),\left(2,20\right),\left(3,15\right),\left(4,10\right),\left(5,8\right),\left(0.5,12\right),\left(0.8,4\right),\left(1,5\right)\}\;\mathrm{Hz}$ (the first number stands for the low cutoff frequency and the second stands for the high cutoff frequency).

Let $F_{l,h}$ denote a band-pass operator applied channel-wise. The filter-bank test-time augmentation can be written as

(3) pl,ht=FFl,hX;θ,  pfil¯t=1B∑l,h∈Bpl,ht,  τfil^=arg maxt pfil¯t.

We then apply the rotation augmentation, which rotates the three-component waveform around each axis a∈{Z,N,E} by angles Φ = {45°, 90°, 135°, 180°, 225°, 270°, 315°}. Let $R_{a}\left(\varphi \right)\in R^{3\times 3}$ be the right-handed rotation matrix about axis a. Using the convention N≡x, E≡y, Z≡z, the three elementary rotations are

(4) $$R_{Z}\left(\varphi \right)=\left[\begin{array}{ccc} \cos\varphi & -\sin\varphi & 0\\ \sin\varphi & \cos\varphi & 0\\ 0 & 0 & 1 \end{array}\right],\;\;R_{N}\left(\varphi \right)=\left[\begin{array}{ccc} 1 & 0 & 0\\ 0 & \cos\varphi & -\sin\varphi \\ 0 & \sin\varphi & \cos\varphi \end{array}\right],\;\;\begin{array}{c} R_{E}\left(\varphi \right)=\left[\begin{array}{ccc} \cos\varphi & 0 & \sin\varphi \\ 0 & 1 & 0\\ -\sin\varphi & 0 & \cos\varphi \end{array}\right]. \end{array}$$

For each sample t, define the rotated data $X^{\left(a,\varphi \right)}\left(t\right)=R_{a}\left(\varphi \right)X\left(t\right)$. The rotation ensemble and can be written as

(5) pa,φt=FXa,φ;θ, prot¯t=13Φ∑a∈{Z,N,E}∑φ∈Φpa,φt,τrot^=arg maxt prot¯t

In the QC module, we quantify prediction consistency by measuring the standard deviation of arrival times across the three TTA outputs and the proportion falling within a narrow time window. By combining diverse augmentations and their deviations, our pipeline achieves high automatic picking accuracy while transparently identifying lower-confidence cases for expert validation.

## 3. Results

Our study first examines how test-time augmentation affects the phase picking results. We then evaluate the impact of the quality control on the credibility of the obtained results. Together, these analyses offer beneficial references for optimizing the reliability of phase picking results.

### 3.1. Results on Test-Time Augmentations

**Figure 3:**
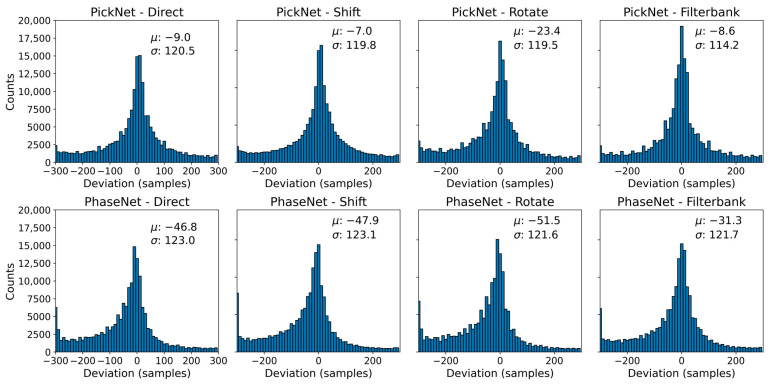
Distribution of sample-level picking errors of PickNet and PhaseNet under four inference settings. Histograms show manual minus automatic deviations (samples; 100 Hz) for (from left to right) direct prediction, time-shift TTA, rotation TTA, and filter-bank TTA. Each panel reports the sample mean (μ) and standard deviation (σ).

Figure 3 shows the distributions of sample-level picking errors (manual minus automatic) for four configurations: direct prediction (no augmentation), random-shift TTA, filter-bank TTA, and rotation TTA for the PickNet and PhaseNet models. The baseline and random-shift TTA exhibit very similar behavior, with mean errors of −9.0 and −7.0 samples and standard deviations of 120.5 and 119.8 samples, respectively, for the PickNet model. Rotation TTA introduces a slight systematic bias: although the standard deviation decreases marginally from 120.5 to 119.5 samples, the mean shifts from −9.0 to −23.4 samples, indicating a less balanced error distribution. By contrast, filter-bank TTA—ensembling predictions across multiple frequency bands—modestly narrows the error spread relative to the baseline and draws a larger fraction of picks into the ±50-sample window; visually, the density of predictions near zero error increases appreciably. The results for the PhaseNet model show similar trends with that of the PickNet model. Under our model and dataset settings, these results suggest that frequency-domain augmentation is particularly effective for improving high-precision, small-error picks. The limited impact of random-shift TTA likely reflects the use of temporal shifts during training, which makes the model’s predictions largely invariant to test-time shifts and therefore similar to the direct (no-augmentation) outputs.

**Figure 4:**
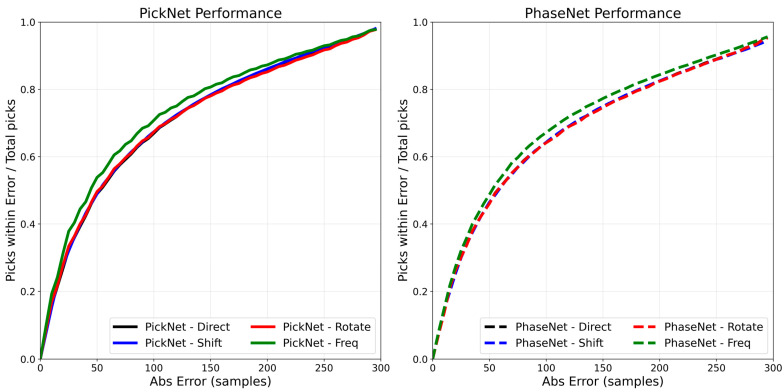
Cumulative accuracy of phase picks under four inference settings. Curves show the fraction of picks with absolute error below a given threshold (*x*-axis, samples at 100 Hz; e.g., 50 samples equal to 0.5 s, 100 samples equal to 1.0 s) for direct prediction (red), time-shift TTA (blue), rotation TTA (gray), and filter-bank TTA (green).

Figure 4 quantifies these trends by plotting, for the PickNet model, the cumulative fraction of picks whose absolute error is below a given threshold (0–200 samples). The filter-bank augmentation consistently dominates the other curves once the threshold exceeds ~20–30 samples, with the largest separation in the mid-range (~30–120 samples), indicating a reduction in moderate errors. The random-shift variant tracks the baseline closely, offering at most a modest uplift across most thresholds, while the rotation ensemble provides no clear benefit and is slightly below the baseline. As the threshold approaches 200 samples, all methods converge toward a similar asymptote, consistent with filter-bank augmentation primarily suppressing moderate outliers rather than changing the extreme tail behavior. Collectively, these curves support the view that frequency-domain augmentation improves effective robustness, whereas simple temporal shifts yield limited incremental gains, and rotation can be slightly counterproductive under the present model and data conditions. The PhaseNet model shows very similar results to that of the PickNet model.

### 3.2. Results on Quality Control Measures

**Figure 5:**
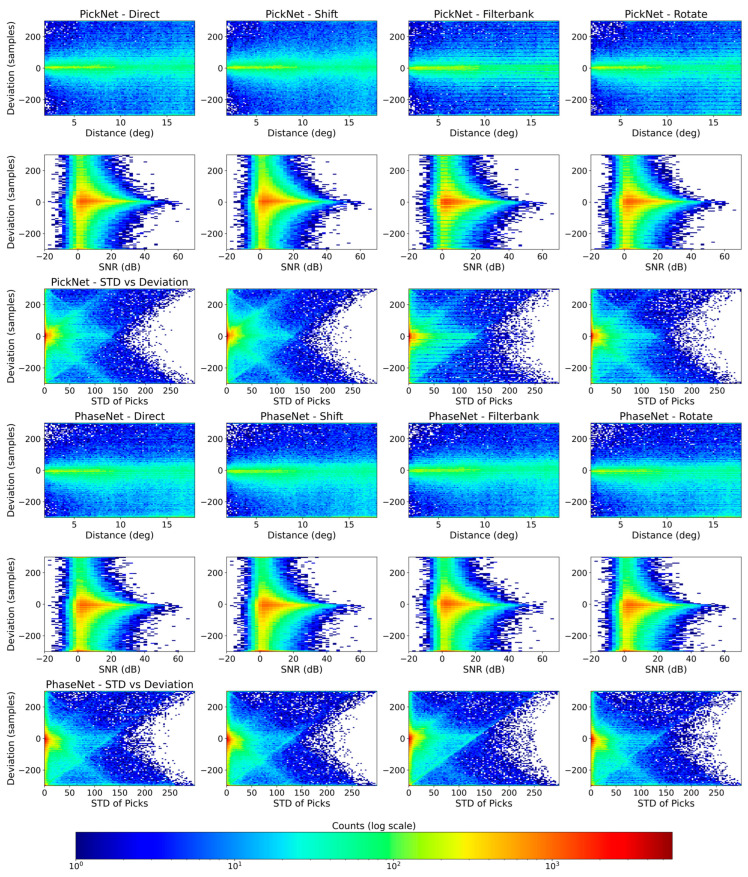
Error behavior versus distance, SNR, and cross-augmentation consistency for four inference settings. Columns correspond to Direct (no TTA), Shift TTA, Filter-bank TTA, and Rotation TTA. Rows show 2-D histograms of manual–automatic deviations (y-axis; samples at 100 Hz) as a function of (first) epicentral distance (degrees), (second) vertical-component SNR (dB), and (third) the standard deviation of picks across the direct and three TTA outputs (“STD of DEV”) for the PickNet model. The fourth to the sixth rows are the same except for the PhaseNet model.

**Figure 6:**
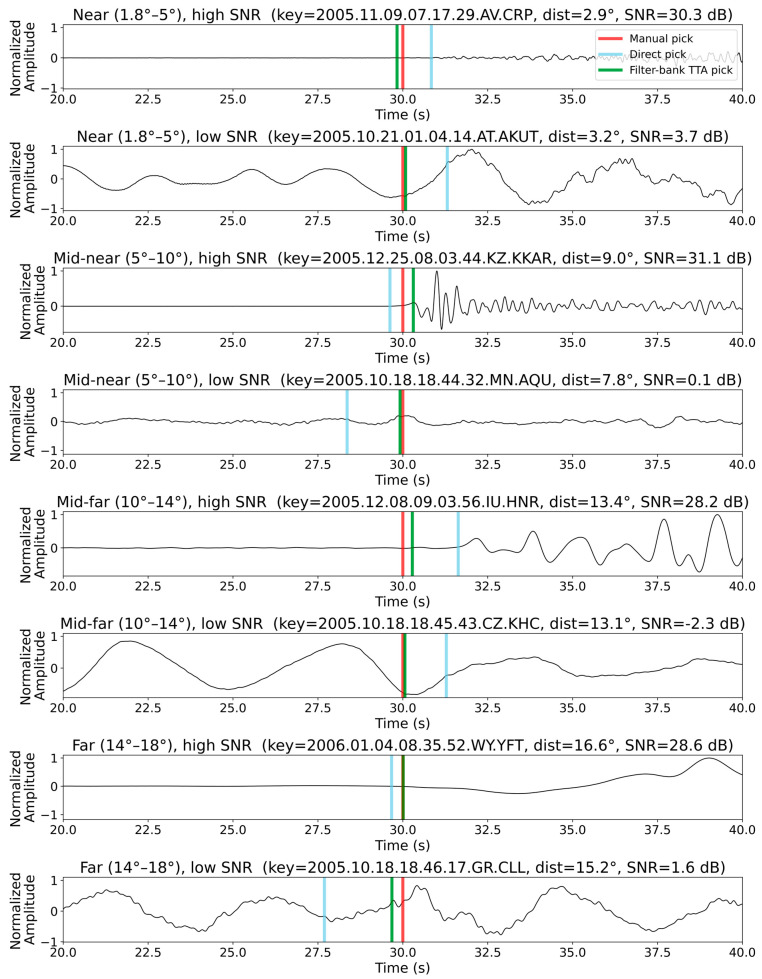
Example Z-component Pn waveforms illustrating automated phase picking for eight events spanning a range of epicentral distances and noise levels. Each panel shows the normalized waveform together with the manual pick (red line), the direct pick by PickNet (blue line), and the pick obtained after filter-bank TTA (green line).

To explore how pick errors relate to epicenter distance, signal-to-noise ratio (SNR), and pick consistency under different augmentations, we computed 2-D histograms of the sample-level deviations (manual minus automatic) against epicentral distance, vertical-component SNR, and the standard deviation of the direct pick and three TTA-augmented picks for both the PickNet and the PhaseNet models. Figure 5 displays these histograms for each of our four methods. For the PickNet model, in the direct baseline (no TTA) panel, error variance grows steadily with distance: picks beyond ~10° show a broadening of both positive and negative deviations. The filter-bank TTA noticeably compresses this distance-dependent scatter toward zero, especially between ~10° and 18°, whereas the rotation ensemble yields a comparatively wide, nearly distance-invariant band, consistent with reduced robustness. The SNR panels display the expected V-shaped pattern: low-SNR records (≤0–5 dB) are associated with large positive and negative deviations, while higher SNRs concentrate density near zero error. Similar patterns are also observed for the PhaseNet model (Figure 5). Please note that for both manual and deep-learning methods, picking arrivals from low-SNR data is difficult, and results in these cases should be interpreted with extra care. Because the Seis-PnSn dataset uses expert-reviewed picks from ISC-EHB, the picks and corresponding errors under low SNR may still be considered reliable. Both random-shift and filter-bank TTAs sharpen this relationship, most prominently in the 10–30 dB range, by collapsing the high-error tails. Rotation again offers little improvement and, in some bins, slightly worsens dispersion relative to the baseline. The consistency analysis demonstrates that the standard deviation across the direct and TTA picks is a strong reliability proxy: higher cross-augmentation dispersion corresponds to larger absolute deviations. Low-STD cases cluster tightly around zero, suggesting that a simple threshold (e.g., STD ≤ 50 samples) can retain a high-confidence subset. Figure 6 shows examples of automated phase picking for data with different epicentral distances and noise levels for an intuitive view.

Building on these insights, we applied a QC filter that retains only picks whose four-way standard deviation falls below 50 samples. Figure 5 plots, for each method, the fraction of retained picks whose absolute error lies within increasing sample thresholds (0–300 samples). QC truncates the long error tail, yielding a noticeably steeper curve: at ±50 samples the rate jumps from 48.98% to 63.16%, and at ±100 samples from 66.94% to 78.46% for the direct phase picking results of the PickNet model. Because the QC statistic relies only on agreement among model variants, it does not require ground truth and thus remains applicable to unlabeled data. The performance convergence across different TTAs is because it preferentially retains stable, consistent picks. With the QC threshold of STD < 50 samples, 75.7% of the picks are retained for PickNet. This retention rate varies only slightly across the different augmentation types, because the QC criterion explicitly selects arrivals whose predicted times are consistent under augmentation. For new augmentation schemes, it is recommended to first visualize the STD distribution and then choose an appropriate threshold based on these diagnostics.

## 4. Usage of the Augmentation Pipeline

Here we provide a pipeline with test-time augmentations to pick first P-arrivals within a user-defined rectangular region and magnitude threshold, then evaluate arrival-time robustness via test-time augmentation (TTA). Using the IRIS FDSN client, the pipeline first queries an event catalog within specified spatial-temporal bounds and retrieves co-located stations limited to three-component HH*/BH* channels. For each event-station pair, theoretical travel times for the target phase (default Pn, TauP/iasp91) are computed from epicentral distance; continuous three-component waveforms are then downloaded for a ±16 s window around the predicted onset, merged, trimmed, and uniformly resampled to 100 Hz to yield a 32 s, 3200-sample tensor. The inference stage feeds this tensor to the deep learning model for four times: (i) direct prediction; (ii) time-shift TTA using symmetric sample offsets with per-shift normalization and window-aligned probability stacking; (iii) band-pass TTA via a pre-defined set of bandpass filters; and (iv) rotation TTA that generates synthetic orientations by rotating the 3-D motion vectors about the Z, N, and E axes across a set of degrees. For each TTA family, class probabilities are aggregated in the 13–19 s subwindow, and the pick is taken at the global maximum of the averaged curve; the script also records the direct pick and the theoretical arrival time. Results are serialized with comprehensive metadata (event, station, back-azimuth, phase, and all pick indices), enabling downstream analysis and optional visualization. Please refer to the Data Availability Statement to access the script.

## 5. Discussion

This work examines how simple test-time augmentations and quality control influence the reliability of deep-learning seismic phase pickers with the Seis-PnSn dataset. Among the tested strategies, frequency filtering augmentation offers the most consistent benefit. It suppresses moderate errors and pulls more picks into tight tolerance windows, with measurable gains at ±0.5–1.0 s before and after QC (Table 1). Temporal shifts yield only small direct improvements, likely because similar jitter is already used during training, making predictions largely invariant to test-time shifts, but they remain valuable as part of a consensus ensemble. Rotation augmentation provides little benefit and can introduce bias for models that were not trained with such transformations or for channel-restricted configurations of PickNet.

The quality control results show that agreement across different augmentations is an effective reliability proxy. The standard deviation of pick times computed from the direct and TTA outputs correlates strongly with absolute error (Figure 5). A simple QC threshold (e.g., <50 samples at 100 Hz) removes long-tail outliers and steepens cumulative accuracy curves across all methods, bringing post-QC performance to a similar, high-confidence level (Table 1). This statistic does not require ground truth, making it suitable for large unlabeled archives where manual picks are unavailable. The proposed TTA plus workflow can serve as a practical baseline for deployment and be helpful for obtaining high-confidence picks.

Error variance increases with epicentral distance and decreases with SNR (Figure 5). The observed SNR sensitivity suggests that denoising methods (e.g., [33]) that could raise effective SNR before inference may also be a promising time-time augmentation. In this study, we do not integrate an explicit denoiser as an additional TTA step. Instead, we treat band-pass filtering as a simple form of denoising because it improves the SNR of the input waveforms. Exploring more advanced denoising methods within the TTA framework is an interesting direction for future work. Another avenue for improvement could be test-time adaptation methods (e.g., [34]), which is worth considering in future works. Although the Seis-PnSn dataset is compiled from global data, its spatial distribution is not uniform. When applying the proposed workflow to a specific region, we recommend collecting a small amount of local data to evaluate performance and, if needed, to conduct regional sensitivity tests. The proposed workflow may be extended to other seismic phases, as they can also benefit from augmentations such as band-pass filtering. However, the choice of filters should be adjusted for different phases. In addition, the effectiveness of shift and rotation tests depends on the data augmentations used during model training.

## 6. Conclusions

We conducted an evaluation of test-time augmentation and quality control for regional seismic phase picking with PickNet and PhaseNet on the Seis-PnSn dataset and obtained proper and practical performance gains compared to the direct inference. Rather than introducing a new picking architecture, our goal was to assess how much existing deep-learning pickers can be improved at inference time by TTA and QC. Filter-bank ensembling reduced picking errors relative to a no-augmentation baseline, while the consistency-based QC via cross-augmentation standard deviation further improved reliability by filtering unstable cases and can be applied even when ground truth is unavailable. In addition, our statistics quantify how performance on Seis-PnSn varies with epicentral distance and SNR and show that filter-bank TTA partly mitigates the degradation at large distances and low SNR, thereby providing practical guidance for applying the workflow and motivating the exploration of denoising methods in particularly noisy settings. Overall, the combination of TTA with QC offers more reliable automated picks without retraining, thereby serving as a practical reference for improving the precision and trustworthiness of automated regional seismic phase picking.

## Figures and Tables

**Table 1:** Performance of different test augmentations before and after the quality control for PickNet and PhaseNet.

Deep Learning Model	TTA-Method	Tolerance (s)	Original	After QC	Tolerance (s)	Original	After QC
PickNet	Direct	0.5	0.4898	0.6316	1.0	0.6694	0.7846
PickNet	Filter TTA	0.5	0.5387	0.6363	1.0	0.7082	0.7856
PickNet	Shift TTA	0.5	0.4893	0.6286	1.0	0.6745	0.7858
PickNet	Rotate TTA	0.5	0.4965	0.6336	1.0	0.6739	0.7853
PhaseNet	Direct	0.5	0.4632	0.5303	1.0	0.6428	0.7189
PhaseNet	Filter TTA	0.5	0.4876	0.5434	1.0	0.6719	0.7308
PhaseNet	Shift TTA	0.5	0.4650	0.5342	1.0	0.6430	0.7189
PhaseNet	Rotate TTA	0.5	0.4607	0.5272	1.0	0.6414	0.7182

## Data Availability

The Seis-PnSn dataset used in this study is publicly available at 10.12197/2023GA018. The pipeline script and the limited PickNet model can be found at 10.5281/zenodo.17303467.

## Abbreviations

The following abbreviations are used in this manuscript:

TTA	Test-time augmentation
QC	Quality control
SNR	Signal-to-noise ratio
